# Critical review of chronic toxicity testing approaches with the saltwater mysid (*Americamysis bahia*) used in pesticide registration

**DOI:** 10.1093/etojnl/vgaf036

**Published:** 2025-02-04

**Authors:** Margaret Fleming, Jane Staveley, Alan Samel, John Aufderheide, T Michelle Blickley, Audrey Bone, Eric Bruns, Tara Catron, Daniel Edwards, Sean Gallagher, Maike Habekost, Cliff Habig, Kevin Henry, Alan Jones, Gwendolin Kraetzig, Shari Long, Patricia Lopez-Mancisidor, Joseph Marini, Amanda Milligan, Adric Olson, Bridget F O’Neill, Eric Peterson, Lee Sayers, Suzanne Schneider, Katie Stump, Seamus Taylor, Theodore Valenti

**Affiliations:** Exponent, Inc, Natick, MA, United States; Exponent, Inc, Chapel Hill, NC, United States; FMC Corporation, Philadelphia, PA, United States; Eurofins EAG Agroscience, Easton, MD, United States; Corteva Agriscience, Indianapolis, IN, United States; Bayer, Leverkusen, North Rhine-Westphalia, Germany; Bayer, Leverkusen, North Rhine-Westphalia, Germany; BASF, Research Triangle Park, NC, United States; BASF, Research Triangle Park, NC, United States; Eurofins EAG Agroscience, Easton, MD, United States; BASF, Ludwigshafen, Rhineland-Palatinate, Germany; Compliance Services International, Lakewood, WA, United States; Tessenderlo Kerley, Inc, Phoenix, AZ, United States; BASF, Research Triangle Park, NC, United States; ISK Biosciences Europe N.V, Diege, Belgium; Valent, San Ramon, CA, United States; Corteva Agriscience, Madrid, Spain; Smithers, Wareham, MA, United States; Eurofins EAG Agroscience, Easton, MD, United States; Envu, Cary, NC, United States; Corteva Agriscience, Indianapolis, IN, United States; Syngenta, Basel, Basel-Stadt, Switzerland; Smithers, Wareham, MA, United States; Eurofins EAG Agroscience, Easton, MD, United States; CropLife America, Arlington, VA, United States; Adama Agricultural Solutions UK, Cologne, United Kingdom; Syngenta, Basel, Basel-Stadt, Switzerland

**Keywords:** aquatic invertebrates, aquatic toxicology, marine toxicity tests, pesticide regulation

## Abstract

The United States Environmental Protection Agency (USEPA) has a conditional requirement for a chronic toxicity test with mysids (*Americamysis bahia*) for registration of pesticide products. Achieving performance acceptability criteria in control treatments for this study can be challenging because the current draft test guideline, which was published in 1996 under USEPA Office of Prevention, Pesticides, and Toxic Substances (OPPTS) 850.1350, Mysid Chronic Toxicity Test, provides limited information on study design and conduct. This critical review was undertaken to (1) identify areas of inconsistency in acceptability criteria between the 1996 draft OPPTS test guideline and ASTM International test guidance, (2) highlight areas that require additional clarification, (3) discuss areas that are impractical or have uncertain scientific relevance regarding the objectives of the test, and (4) provide recommendations for revision of the draft OPPTS test guideline. To achieve this, 116 final study reports from chronic mysid toxicity tests conducted over approximately the past 30 years were collected. From these reports, survival, growth, and reproduction data from negative and solvent control groups were compiled. Through investigation of trends in the data, it became apparent that no-observed-effect concentrations (NOECs) were most commonly based on reproductive endpoints, followed by adult growth endpoints and adult survival. Notably, less than 1% of studies had a NOEC based solely on a second-generation measurement endpoint. Analysis of this comprehensive data set provided clarity on the establishment of acceptability criteria for the study and elements of the testing procedure that can be streamlined without loss of critical information, which resulted in a set of recommendations to improve future versions of the chronic mysid toxicity test guideline.

## Introduction

**Figure 1.**
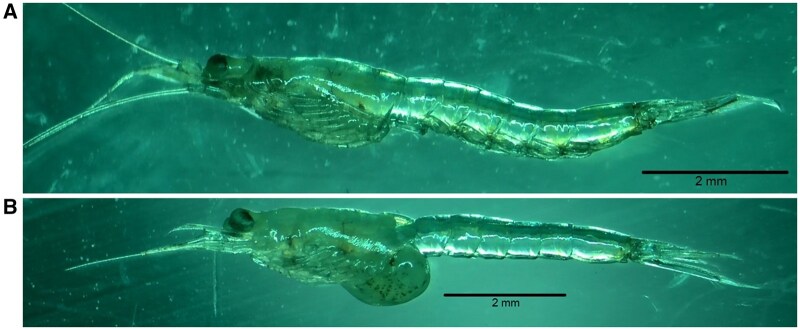
(A) Photograph of male *Americamysis bahia*. (B) Photograph of gravid female *Americamysis bahia*.

Mysids are aquatic crustaceans colloquially known as “opossum shrimp” for their shrimp-like appearance and brood pouch (Figure 1). The marine mysid species *Americamysis bahia* (formerly *Mysidopsis bahia*) is used as a test organism due to its availability, sensitivity to toxic chemicals, short life cycle, and ability to be cultured in the laboratory (Anderson & Phillips, 2016; Nimmo & Hamaker, 1982). Chronic mysid toxicity testing is required by the United States Environmental Protection Agency’s (USEPA) Office of Pesticide Programs, within the Office of Chemical Safety and Pollution Prevention (OCSPP), formerly known as the Office of Prevention, Pesticides, and Toxic Substances (OPPTS), for the registration of pesticide products in the United States if pesticide residues from the proposed uses can potentially enter waterways (40 CFR § 158.630). The mysid chronic toxicity study is not typically required for pesticide registration in countries or regions outside of North America (e.g., chronic mysid testing is not required according to European Union [EU] data requirements as laid down in Commission Regulation (EU) No. 283/2013). In most countries, chronic aquatic invertebrate risk assessments are performed using endpoints derived from the freshwater *Daphnia magna* chronic toxicity study (Organisation for Economic Co-operation and Development [OECD] 211; OECD, 2012) or OCSPP 850.1300 (OCSPP, 2016). However, some regulatory agencies will use a mysid study endpoint to perform their risk assessments in place of the chronic *Daphnia magna* endpoint if a mysid study is available and submitted, particularly if the endpoint is lower than that of the chronic *Daphnia magna* study.

The USEPA published the *OPPTS 850.1350 Mysid Chronic Toxicity Test* draft guideline in 1996 (OPPTS, 1996). (USEPA guidelines published prior to April 22, 2010, retain the numbering convention adopted in 1994 specifically including OPPTS as part of the guideline’s number.) The purpose of the mysid chronic toxicity test as stated by the draft OPPTS test guideline is to “develop data on the chronic toxicity of chemicals ….[and] use data from these tests in assessing the hazard of a chemical to the aquatic environment.” The purpose of the definitive test is to determine “effects of a chemical on growth and reproduction during chronic exposure.” To reach this determination, mysids are exposed continuously to five or more concentrations of the test chemical for a period of 28 days. Tests also include control groups (negative and solvent, if applicable) in which mysids are exposed to the same conditions as the mysids in the treatment groups without the addition of the test chemical. A minimum of 40 mysids per group (control and treatment groups) are used. As stated by the draft OPPTS test guideline, at sexual maturity (approximately 10–14 days after the start of the exposure), mysids are separated into replicate groups of no more than eight mysids each. As second-generation (G2) offspring are produced, they are counted and separated into retention chambers at the same test substance concentration as the chambers where they originated. Multiple measurement endpoints are reported, as available, including the number of dead mysids in each chamber on Days 7, 14, 21, and 28, number of male and female mysids in each test chamber when sexual characteristics become apparent, body length of males and females at sexual maturity and test termination, number of offspring, and offspring mortality.

The draft OPPTS test guideline, while listed as conditionally required, has not been updated by USEPA’s pesticide program office since being drafted in 1996. The current draft OPPTS test guideline provides limited technical information on study design and study conduct, and achieving the described test organism performance criteria is difficult. A joint CropLife America-CropLife Europe (CLA-CLE) workshop was held July 14, 2020, to establish a working group to review the draft OPPTS test guideline study design and subsequently develop recommendations for the modernization of the test guideline. This process included reviewing both the draft OPPTS test guideline and guidance published by ASTM International on conducting life-cycle toxicity tests with saltwater mysids—E1191-03 Standard Guide for Conducting Life-Cycle Toxicity Tests With Saltwater Mysids (ASTM International, 2003). The focus of this review was on the draft OPPTS test guideline, although information from the ASTM International test guideline was also considered.

The purpose of this critical review, a product of the CLA-CLE working group, is to (1) identify areas of inconsistency in acceptability criteria between the draft OPPTS test guideline and the ASTM International test guideline, (2) highlight areas in the draft OPPTS test guideline that require additional clarification, (3) discuss elements of the draft OPPTS test guideline that either lack practicality or have uncertain scientific relevance with respect to the objectives of the test, and (4) provide recommendations for revision of the draft OPPTS test guideline.

## Methods

Pesticide companies in the CLA-CLE working group provided final study reports of mysid chronic toxicity studies conducted with a variety of pesticide active ingredients. Results from the collected studies were anonymized with respect to sponsor, testing facility, and active ingredient prior to entry into the data set.

### Study-specific information collection

For each study, information was collected to populate the following fields. These were exposure duration, number of control replicates, number of pairing chambers, G2 exposure duration, and the most sensitive measurement endpoint.

The most sensitive measurement endpoint refers to the measurement endpoint (e.g., survival, reproduction, or growth) that displayed a statistically significant difference from the control group at the lowest concentration (i.e., lowest-observed-effect concentration [LOEC]). The most sensitive measurement endpoint determines the no-observed-effect concentration (NOEC), defined as the test concentration immediately below the LOEC. Lowest-observed-effect concentration and NOEC values were those identified within the final study reports based on comparison to the negative control (if no solvent was used), the solvent control, or the combined controls and were not independently recalculated.

### Frequency analysis of most sensitive measurement endpoints

A frequency analysis to determine the most sensitive measurement endpoints (e.g., those determining the NOEC) was performed. For this analysis, the reproductive endpoints (e.g., number of young produced per surviving female, percentage of females producing offspring, day of first brood release) were categorized together, and the growth endpoints (body wt, body length) were categorized together (Table 1). The G2 endpoints were categorized separately from adult (G1) endpoints. If multiple categories demonstrated the same level of sensitivity (i.e., the same NOEC), they were represented as a combined category. For example, if number of young produced per surviving female (reproduction) and male length at test termination (growth) displayed the same NOEC, the most sensitive measurement endpoint category for the study would be “reproduction and growth.” For another example, if G1 survival and G2 survival displayed the same NOEC, the most sensitive measurement endpoint category would be “survival and G2 survival.” This was done to prevent overweighting endpoints when other endpoints in the study were equally sensitive and to prevent overweighting studies with several equally sensitive endpoints (e.g., double counting a study with two equally sensitive endpoints). Studies that did not find a significant impact on any measured endpoint were not included in the frequency analysis.

**Table 1.** Endpoint categories for sensitivity analysis.

Endpoint category	Endpoint
Survival	Survival
Reproduction	Day of first brood release
	Number of brood releases
	Young produced per reproductive day
	Young produced per surviving female
Growth	Male body weight at test termination
	Female body weight at test termination
	Male body length at test termination
	Female body length at test termination
	Male body length at sexual maturation
	Female body length at sexual maturation
G2 Survival	G2 Survival
G2 Growth	Male body length at test termination
	Female body length at test termination

*Note.* G2 = second generation.

### Control group data aggregation and analysis

Data for negative and solvent control groups (where a solvent control was used) were both separately included in the data set for the current analysis (47% of studies had both negative and solvent controls). For each control group, results for the following fields were collected where data were available, and the average value was calculated. Unless specified as G2, all measurement endpoints relate to G1. These fields were control type (i.e., negative or solvent; if a study reported negative and solvent control results, they are both included as separate entries), survival (%), day of first brood release, number of brood releases, young produced per reproductive day (number of reproductive days is defined as the number of days starting from the earliest day of brood release of any replicate in any control or treatment to the total number of days in the study), young produced per surviving female, male body weight at test termination (mg), female body weight at test termination (mg), male body length at test termination (mm), female body length at test termination (mm), male body length at sexual maturation (mm), female body length at sexual maturation (mm), G2 survival (%), G2 male body length (mm), and G2 female body length (mm).

The average values calculated for each control group were plotted to show the distribution of measurement endpoint values. Additional statistical values were calculated to characterize the distribution including quartiles, average, standard deviation, and coefficient of variation (CV).

## Results

### Data collection

A total of 116 mysid chronic toxicity study reports finalized between 1987 and 2021 were obtained. These final study reports were spread among 18 sponsors representing six individual testing laboratories. The inclusion of both negative and solvent control groups from the final study reports resulted in a total of 170 control sets in the data set.

### Frequency analysis of most sensitive measurement endpoint

**Figure 2.**
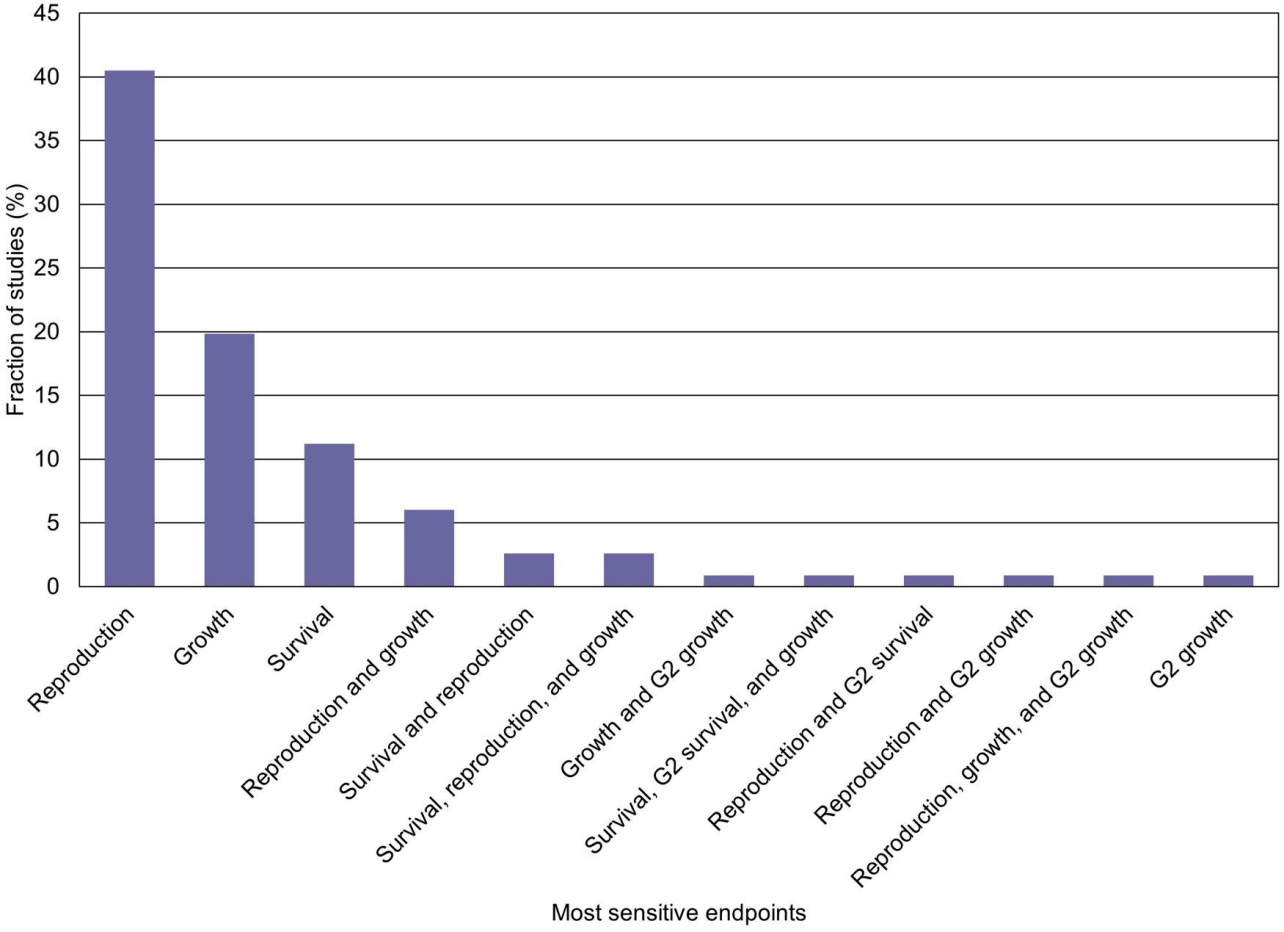
The most sensitive measurement endpoints reported in the assembled mysid chronic toxicity study reports. Unless specified as an exposed offspring (generation 2; G2) of adult generation (G1) endpoint, these endpoints refer to effects on the exposed adult generation. Fourteen of the collected studies are not included because no significant impact on any measurement endpoint was observed.

The most sensitive measurement endpoints from the compiled studies are presented in Figure 2. Reproduction was most commonly associated with study NOECs (41% of NOECs), followed by growth (20% of NOECs), survival (11% of NOECs), and both reproduction and growth (6% of NOECs). The measurement endpoints in these categories were the primary drivers of the resulting NOEC values, accounting for more than 75% of NOECs. Only 1 study of 116 (0.86% of the studies) reported a NOEC based on a G2 endpoint alone, with G2 male length as the most sensitive measurement endpoint.

### Measurement endpoint reporting

**Figure 3.**
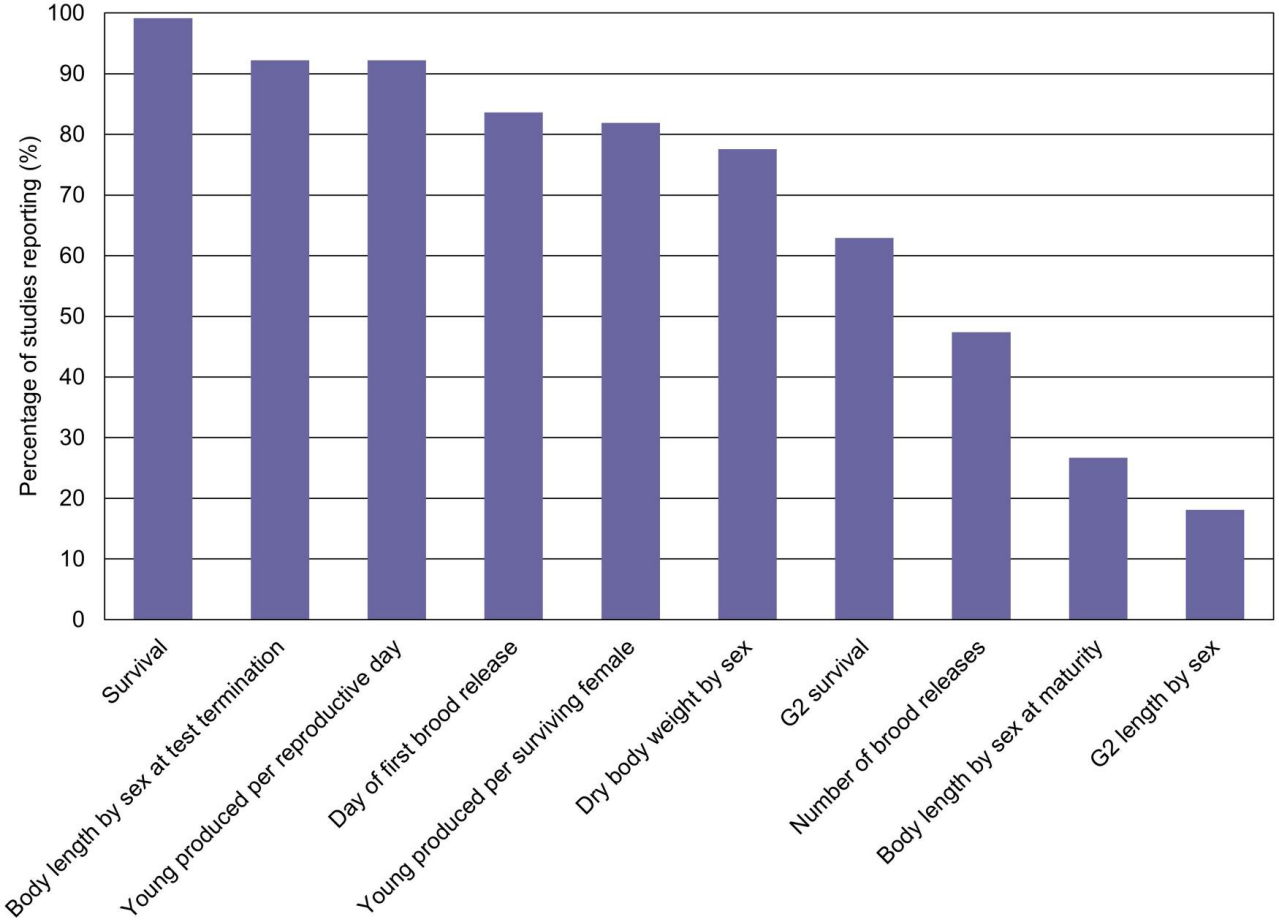
Fraction of studies reporting each considered measurement endpoint. Includes studies that reported enough information to calculate the endpoint if not directly reported.

The specific measurement endpoints reported were not always consistent across studies. Figure 3 presents the fraction of studies that included information on each of the measurement endpoints of interest. In some cases, measurement endpoints that were not included in the study report were calculated for the purposes of this review. Adult exposures typically lasted 28 days (ranging from 28 to 40 days), while G2 exposures, included in approximately two-thirds of the studies, typically lasted 4 days (ranging from 4 to 12 days). The online supplementary material provides the data for each study and the calculated statistical values (i.e., quartiles, average, standard deviation, CV) describing the results from the control groups (including both negative and solvent controls) for all measurement endpoints of interest (see online supplementary material Table S1). Key aspects of the statistical analyses are provided in Table 2 for reference.

**Table 2.** Statistical distribution of control results reported in the collected chronic mysid toxicity studies. Note that the count may exceed the study count (116 total studies) because results from both negative and solvent control groups were included when provided.

Endpoint	Count	Min	25th percentile	Median	75th percentile	Max	Average	Standard deviation	CV (%)
Survival (%)	169	60.3	79.0	87.9	94.0	100	86.3	9.54	11.1
Young produced per reproductive day	157	0.20	0.57	0.96	1.48	2.50	1.06	0.60	56.5
Young produced per surviving female	138	3.26	7.88	13.9	21.8	42.0	15.6	9.05	58.0
Day of first brood release	140	14.9	16.8	18.0	20.3	30.8	19.0	3.31	17.5
Number of brood releases	72	1.17	2.14	2.41	2.72	3.57	2.39	0.52	21.7
Male body length at termination (mm)	153	5.18	6.24	7.35	7.79	9.12	7.17	0.89	12.5
Female body length at termination (mm)	153	5.78	6.45	7.56	7.99	9.44	7.40	0.84	11.4
Male body weight (mg)	128	0.62	0.82	0.91	0.99	1.29	0.91	0.13	14.2
Female body weight (mg)	128	0.74	1.07	1.21	1.32	1.90	1.19	0.20	16.4
G2 survival (%)	101	67.2	90.0	97.5	100	100	94.0	6.80	7.24

*Note.* Min = minimum; Max = maximum; CV = coefficient of variation; G2 = second generation.

### Control survival

**Figure 4.**
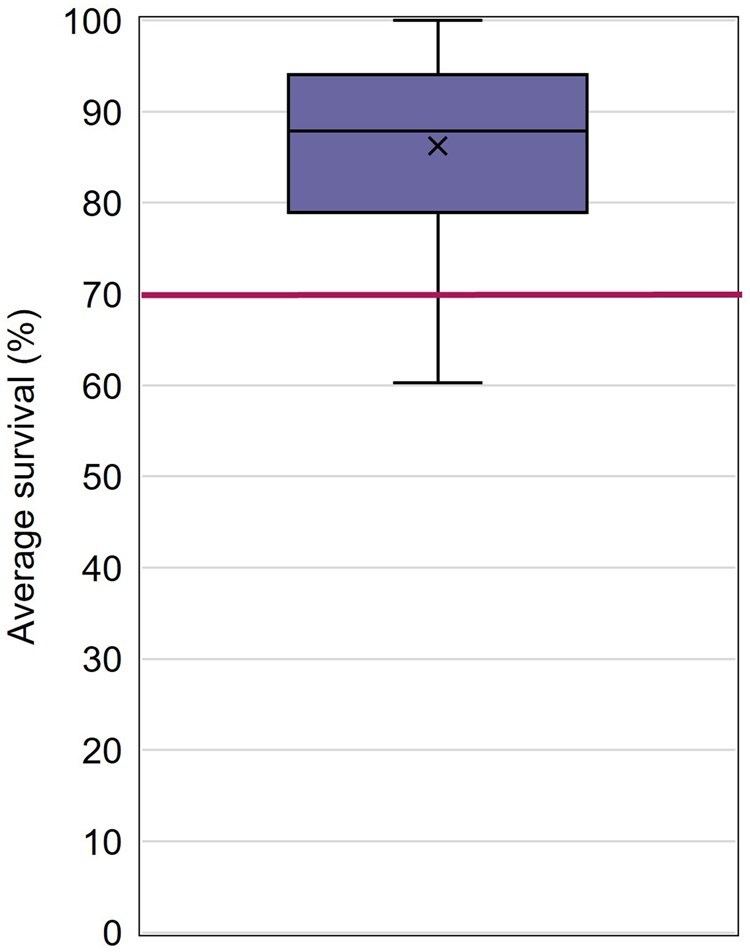
Average adult survival quartile distribution with the average value indicated with an “X.” The horizontal line indicates the ASTM International test guideline acceptability criterion for control survival (70%). *Note*. ASTM = American Society for Testing and Materials.

Average survival in G1 control groups ranged from 60.3% to 100%, with an overall average survival value of 86.3% (CV = 11%; Figure 4, Table 2). Survival was similar in solvent control groups (62.0%–100%; average of 87.3%; median 89.7%) when compared to negative control groups (60.3%–100%; average of 85.8%; median of 86.5%).

### Control reproduction

**Figure 5.**
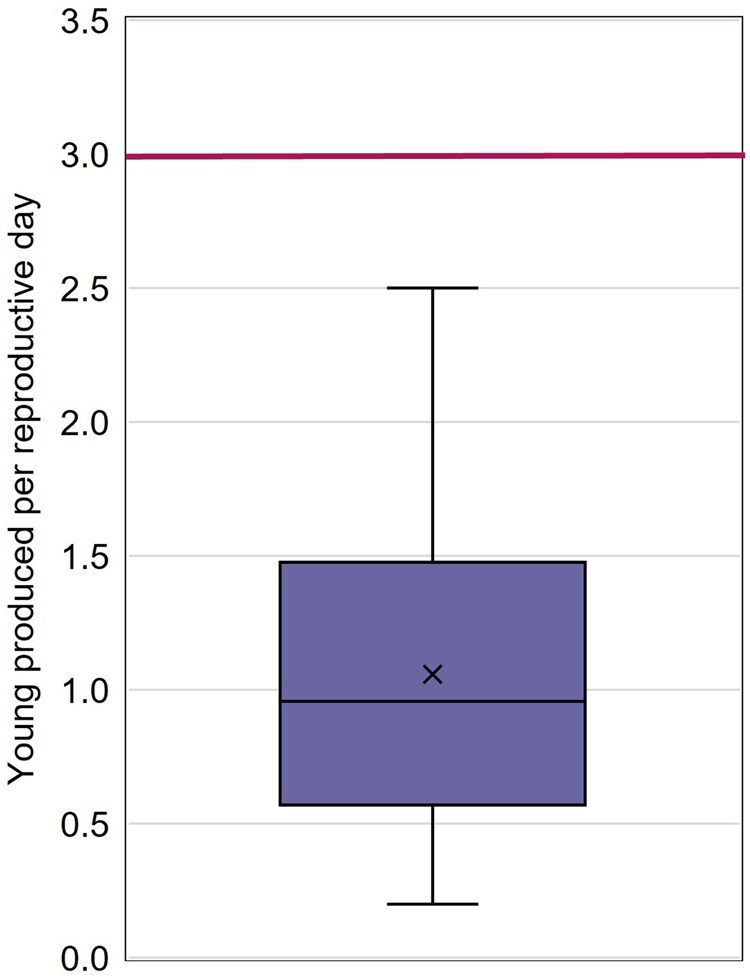
Average number of young produced per reproductive day quartile distribution with the average value indicated with an “X.” The horizontal line indicates the draft Office of Prevention, Pesticides, and Toxic Substances test guideline acceptability criterion for number of offspring per control female per day (3).

**Figure 6.**
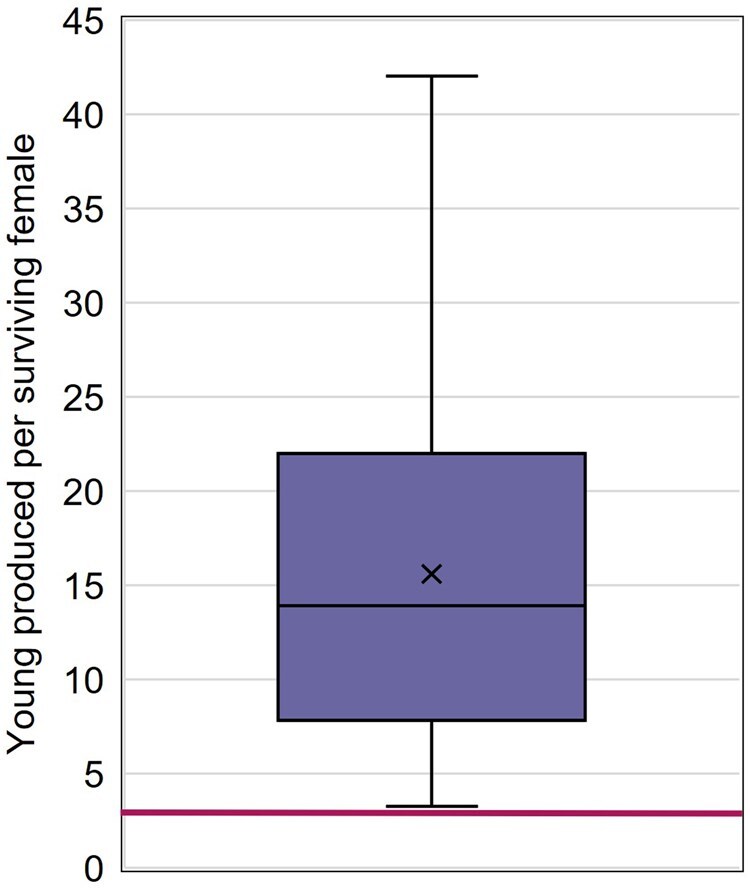
Average number of young produced per surviving female quartile distribution with the average value indicated with an “X.” The horizontal line indicates the ASTM International test guideline acceptability criterion for average number of offspring per control female (3). *Note*. ASTM = American Society for Testing and Materials.

Almost all studies reported the number of young produced per female per day (91.4% of studies) and the number of young produced per surviving female (81.9% of studies; Figure 3). The average number of young produced per reproductive day ranged from 0.20 to 2.50 (Figure 5, Table 2). Across all control groups, the number of young produced per reproductive day averaged 1.06 with a CV of 56.5%. From the collected study control data, the average number of young produced per surviving female ranged from 3.3 to 42 (Figure 6, Table 2). The average number of young produced per surviving female across all control groups was 15.6 (CV = 58.0%).

**Figure 7.**
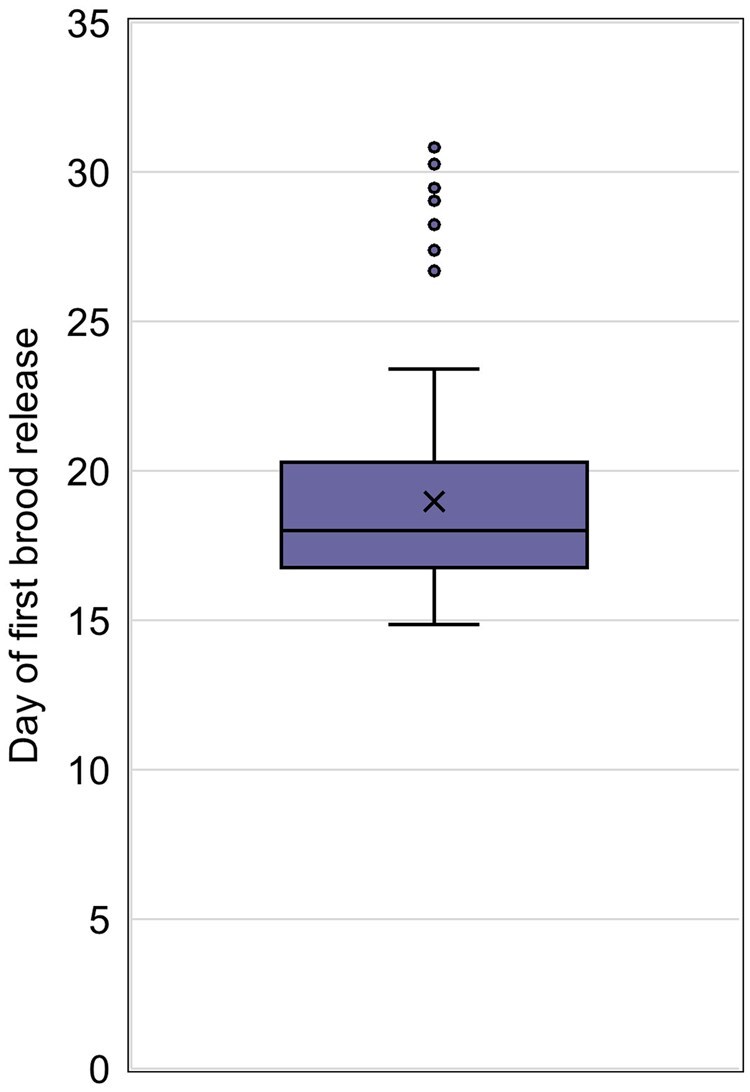
Average day of first brood release quartile distribution with the average value indicated with an “X” and statistical outliers shown as separate points outside of the box and whisker plot.

**Figure 8.**
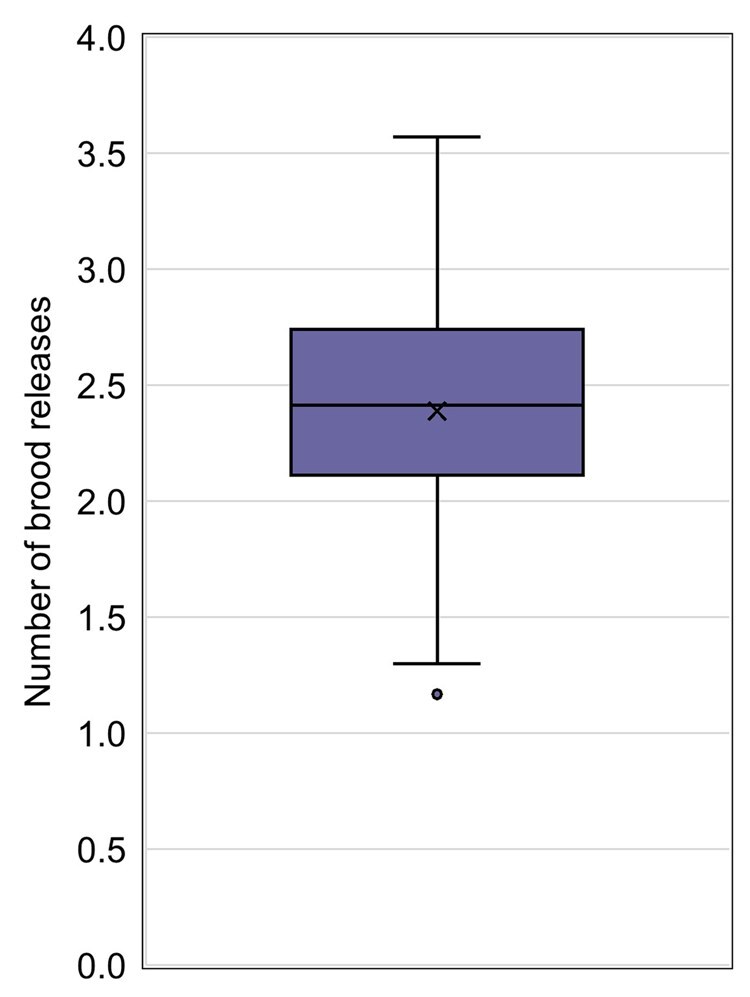
Average number of brood releases quartile distribution with the average value indicated with an “X” and a statistical outlier shown as a separate point outside the box and whisker plot.

The average day of first brood release, included in 83.6% of studies (Figure 3), ranged from 14.9 to 30.8 with an average value across control groups of 19.0 (Figure 7, Table 2). The number of brood releases was less commonly reported, included in 47.4% of studies (Figure 3). The average number of brood releases ranged from 1.17 to 3.57 (Figure 8, Table 2). The CVs associated with the day of first brood release and the number of brood releases were 17.5% and 21.7%, respectively.

### Control growth

**Figure 9.**
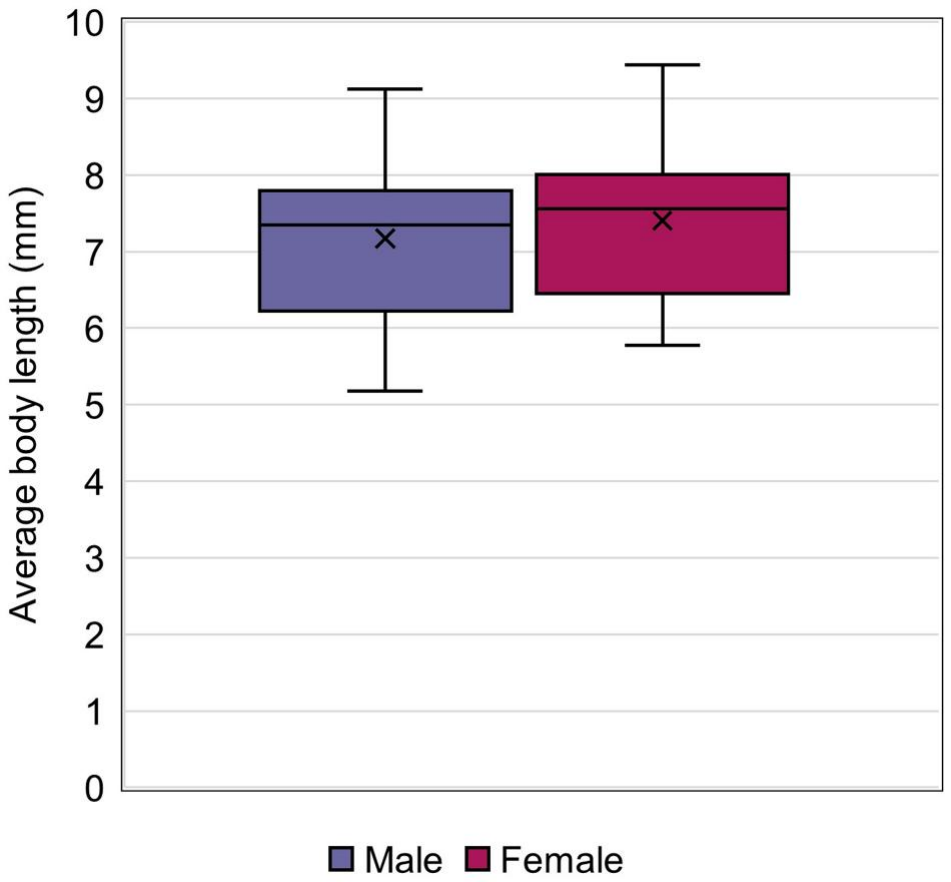
Average body length at test termination quartile distribution with the average value indicated with an “X.”

Average body length at termination was reported in 92.2% of studies (Figure 3), with values ranging from 5.18 to 9.12 mm for males and from 5.78 to 9.44 mm for females (Figure 9, Table 2). The average body length at test termination was 7.17 mm for males (CV = 12.5%) and 7.40 mm for females (CV = 11.4%; Figure 9, Table 2). Because body length at sexual maturity was not included in a majority (73.3%) of the studies, statistics were not calculated for this measurement endpoint.

**Figure 10.**
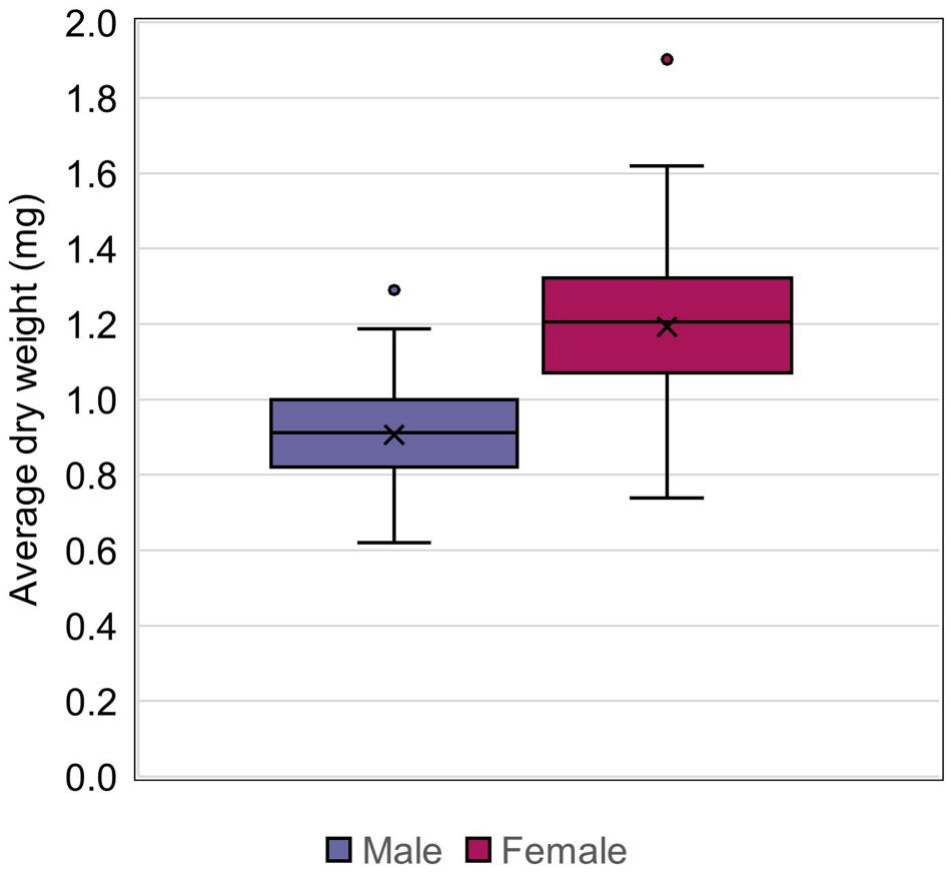
Average dry weight at test termination quartile distribution with the average value indicated with an “X” and statistical outliers shown as separate points outside the box and whisker plots.

Body weight, reported (as dry wt) by sex, was included in 77.6% of the study reports (Figure 3). Average body dry weight at termination ranged from 0.62 to 1.29 mg for males and from 0.74 to 1.90 mg for females (Figure 10, Table 2). The average body dry weight at termination was 0.91 mg for males (CV = 14.2%) and 1.19 mg for females (CV = 16.4%).

### Control G2 measurement endpoints

**Figure 11.**
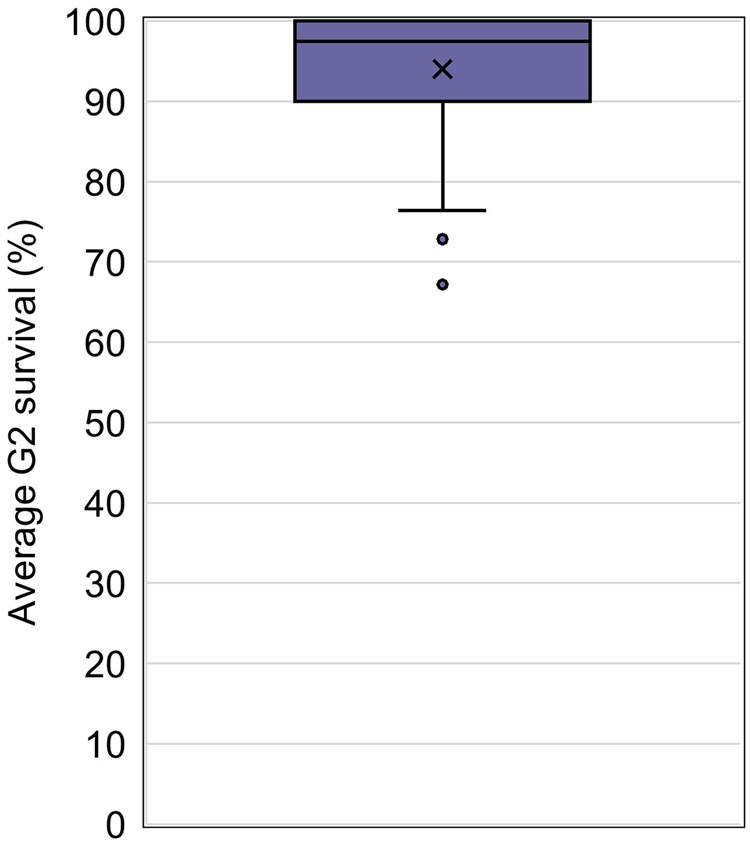
Average G2 survival (%) quartile distribution with the average value indicated with an “X” and statistical outliers shown as separate points outside the box and whisker plot. *Note*. G2 = second generation.

The G2 survival was reported in 63% of studies (Figure 3). Average G2 survival for the control groups was high, ranging from 67.2% to 100% with an overall average value of 94.0% (CV = 7.24%; Figure 11, Table 2). As stated, two-thirds of the G2 exposures were carried out to 4 days, which is not sufficient time for sexual development to occur with this species. Therefore, statistics were not calculated for G2 body length by sex because this measurement endpoint was only included in a minority of the collected studies (18.1%).

## Discussion and recommendations

### Test guideline acceptability criteria

Acceptability criteria in the draft OPPTS test guideline include a test duration of 28 days and two criteria for the reproductive performance of adult female mysids in the control group(s), described as resulting in an unacceptable test if not met. The first reproductive criterion is a requirement that at least 75% of adult female mysids produce offspring. The second reproductive criterion is a requirement that each adult female mysid produces an average of at least three offspring per day. In addition to outlining acceptability criteria, the draft OPPTS test guideline requires reporting of a variety of measurement endpoints. These include the time at which sexual characteristics are discernable for all test organisms (i.e., day of sexual maturation), the number of male and female mysids in each test chamber at sexual maturation, day of first brood release, body length by sex at sexual maturation, body length by sex at test termination, total number of young produced per female at test termination, mortality on Days 7, 14, 21, and 28, G2 mortality, number of G2 males and females, and G2 body length by sex.

A comparison of the control performance criteria stated in the draft OPPTS test guideline and the ASTM International test guideline is provided in Table 3. Consistent with the draft OPPTS test guideline, the ASTM International test guideline acceptability criteria require at least 75% of the adult female mysids in the control group(s) produce offspring. However, the two test guidelines diverge on the required number of offspring produced per female. Whereas the draft OPPTS test guideline requires an average of at least three young to be produced per female per day, the ASTM International test guideline requires at least three young to be produced per female over the full duration of the study. The ASTM International test guideline also requires that at least 70% of the exposed adult mysids survive until test termination, whereas the draft OPPTS test guideline does not include a criterion for control survival. Note that both the draft OPPTS test guideline and ASTM International test guideline have additional specifications and acceptance criteria regarding test conditions and other study reporting elements; these are not discussed here.

**Table 3.** Comparison of control performance criteria provided in the draft Office of Prevention, Pesticides, and Toxic Substances test guideline and ASTM International test guideline.

Parameter	Control performance criteria	
	Draft OPPTS test guideline	ASTM International test guideline
Percentage of surviving adults	No requirement	70%
Percentage of adult females that produce offspring	75%	75%
Rate of offspring production	3 per female per day	3 per female

*Note.* OPPTS = Office of Prevention, Pesticides, and Toxic Substances; ASTM = American Society for Testing and Materials.

### Control survival

Survival of control adults was consistently high in the studies reviewed. While the draft OPPTS test guideline does not include criteria for control survival, the ASTM International test guideline specifies that if more than 30% of the G1 control mysids die between the start and end of the test, the test should not be considered acceptable. Based on average survival per control group, the ASTM International test guideline criterion was achieved by 93% of the studies submitted for this project, indicating that is an appropriate criterion. If a survival criterion is implemented in the revised test guideline, it should allow for the study director to consider the impact of inadvertent mortalities (e.g., impingement, mysids that jump from the retention chamber and desiccate) on the survival calculations.

### Control reproduction

Both the draft OPPTS test guideline and the ASTM International test guideline include acceptability criteria for female mysid reproduction. According to the draft OPPTS test guideline, test results are unacceptable if either more than 25% of females fail to produce young (i.e., at least 75% of females must produce young) or an average of less than three offspring are produced per female per day. The ASTM International test guideline also includes an acceptability criterion of at least 75% of females producing offspring, but it states a study total, rather than daily requirement, of three offspring per female. All studies that reported the percentage of surviving females that produced offspring met the ASTM International test guideline and draft OPPTS test guideline criterion of at least 75% of females producing young. The second reproductive criterion of the test guidelines, based on the rate of offspring production, requires further consideration.

The OPPTS test guideline states that a test is unacceptable “if the average number of young produced per female in the controls is less than three per day.” None of the control groups had an associated average value that met the stated reproductive acceptability criterion of three offspring per female per day. Although not contained in written guidance, this criterion appears to currently be recognized as infeasible and is generally not considered to be a reason to invalidate a study during regulatory review. Data Evaluation Records (DERs) are prepared by USEPA to evaluate the acceptability and utility of studies submitted to the agency. Generally, a DER may note that not meeting this criterion is a guideline deviation, but this does not appear to influence the acceptability of the study by the reviewer. However, because the requirement is listed in the draft guideline, regulatory authorities outside the United States and others may expect this outcome and reject a study when it is not achieved.

The ASTM International test guideline has a less restrictive reproductive criterion, stating that tests are considered unacceptable if “the average number of young produced by the first-generation females in the control(s) was less than three.” It is considered that this refers to the average number of young produced per female surviving until test termination. This ASTM International data quality objective was achieved by all studies in the data set.

Based on the control data from the collected studies, the draft OPPTS test guideline reproductive acceptability criterion related to the offspring production rate is not achievable, not because of any deficiency in the technical conduct of the chronic tests, but due to the wording in the OPPTS test guideline. The ASTM International test guideline reproductive criterion requiring each surviving female to produce, on average, at least three offspring during the test period is a more appropriate performance threshold for control groups. The criterion that both the ASTM International and the draft OPPTS test guidelines include, that 75% of females must produce offspring, is consistently met by control groups.

### Variability in reproduction

The CVs for young produced per reproductive day for individual control groups had a median value of 19.6% and an average value of 22.5%. The CVs for young produced per surviving female for individual control groups had a median value of 20.2% and an average value of 23.5%. While the CVs ranged as high as 84.8%, 75% of control group CVs were less than 35% for both reproductive measurement endpoints. Considerable variation about the mean is expected for reproductive measurement endpoints based on number of offspring. Lussier et al. (1996), who assessed the variability of *A. bahia* reproduction across five different test designs, found that for all five test designs, the CVs associated with the number of young released per female mysid were over 50%.

Agencies in the EU sometimes request the concentration that has an observable effect on 10% of exposed organisms (EC10) values in lieu of NOEC values for pesticide registration. We note, however, that regression-based endpoints from hypothesis-based study designs can be misleading and ECx values should be evaluated relative to the empirical NOEC and LOEC. Effect concentration10 values have been demonstrated to be statistically unreliable for measurement endpoints with significant variability, such as dry weight and shoot height for terrestrial plants, where the minimum detectable difference (MDD) is often well above 10% (Staveley et al., 2018). The MDD relates to the CV; therefore, given the high CV values associated with control reproductive endpoints, EC10 values are not appropriate for the assessment of chronic mysid studies. This should be specified in the test guideline to avoid misinterpretation of results by reviewers who may not be familiar with the natural variability of chronic mysid studies and request determination of endpoints (e.g., calculation of EC10 endpoints) for which the study design is not suitable. It may be appropriate to derive other ECx values, but this depends on an understanding of the inherent variability in the measurement endpoint being analyzed. Further evaluation would be required to determine what ECx level would be appropriate for a given measurement endpoint in this study design.

While there is sometimes significant variability in reproductive endpoints, these measurement endpoints were the primary NOEC drivers in the studies in this analysis (Figure 2). Reproductive endpoints are an important focus of chronic ecotoxicity tests because they provide information to assess potentially population-relevant effects of a test substance.

The statistical variation associated with both the day of first brood release and the number of brood releases is lower than the variation associated with offspring production endpoints as would be expected due to the limitations imposed by the test duration. However, because some laboratories used group housing of paired mysids after sexual maturation, the average number of brood releases was not always available. The day of first brood release might also vary depending on food quality and environmental parameters, such as temperature and season. Therefore, it is recommended that those measurement endpoints are reported but not used as acceptability criteria with threshold values.

### Control growth

While the draft OPPTS test guideline and ASTM International test guideline acceptability criteria are only provided for adult survival and reproduction, the draft OPPTS test guideline also requests that additional measurement endpoint data be reported. The draft OPPTS test guideline instructs that body length should be measured at the time sex can be determined simultaneously for all mysids in control and treatment groups and again at test termination, specified as Day 28.

Body length at sexual maturity was included in a minority (26.7%) of the studies (Figure 3). This lack of inclusion may be related to the difficulty associated with collecting this measurement without unintentionally impacting the test organisms. The draft OPPTS test guideline suggests achieving this mid-exposure measurement without applying undue stress to the test organisms by collecting the data using “photography through a stereomicroscope with appropriate scaling information.” However, this may not be practical or feasible in all studies. Furthermore, study organisms will still likely incur additional stress due to necessary handling such as placement and adjustment under a stereomicroscope. There is no test guideline acceptability criterion associated with body length; thus, it may not be necessary to include body length by sex at sexual maturation as a reporting requirement. Based on our data set, studies are already commonly measuring body length by sex at test termination and control results show high consistency, indicating that this is a reliable reporting endpoint for growth. Given the potential adverse impact on test organisms and the lack of benefits of inclusion, we recommend excluding body length at sexual maturity as a reporting requirement from future test guidelines.

While variability for control female dry weight was low in the data set (CV = 16.4%), it should be noted that female dry weight can be influenced by the presence or absence of young in the brood pouch at the time of test termination. This factor should be considered for studies in which female dry weight at test termination impacts the study NOEC.

### Control G2 performance

The draft OPPTS test guideline requests that G2 measurement endpoints of mortality, number of males and females, and male and female body length be reported “if available prior to termination of the test (day 28).” G2 body length by sex was included in a minority of the reviewed studies (18.1%). Given the 28-day testing window specified by the draft OPPTS test guideline, offspring may not reach sexual maturation by the time of test termination, limiting the ability to differentiate G2 males from G2 females.

While a reporting requirement for G2 mortality and G2 growth is specified in the draft OPPTS test guideline, no acceptability criteria are specified for G2 measurement endpoints in either the ASTM International test guideline or the draft OPPTS test guideline. (The G2 observation is optional under the ASTM International test guideline.) Moreover, the results of this analysis indicate that inclusion of the G2 exposure does not regularly impact the regulatory endpoint, as evidenced by the fact that the G2 measurement endpoints changed the overall study NOEC in only 1 of the 116 study reports (<1%). This single study reported G2 male length as the most sensitive measurement endpoint. The differences between the G2 response at the NOEC and LOEC compared to the pooled control value for this study are 1.9% and 7.4%, respectively. Therefore, it could be concluded that the LOEC is not biologically significantly different than the pooled controls. A significant impact on both adult growth and reproduction (i.e., adult female mysid length and day of first brood release) was observed at the next highest exposure concentration to the concentration affecting G2 male length. Therefore, for the single study with a NOEC based solely on a G2 measurement endpoint, inclusion of the G2 endpoint altered the NOEC value by only one concentration step. The only exposure concentration that resulted in at least a 10% difference in the G2 population response was the highest exposure concentration (17% difference), higher than the non-G2 NOEC for this study.

Based on our review of 116 chronic mysid studies, observations made during the G2 phase of exposure appear ineffective and insensitive with respect to endpoint identification, and this phase also disproportionally and unnecessarily increases complexity and handling efforts. The results of the G2 exposures impact study results so rarely that their exclusion from future test guidelines will not have a significant effect on test outcomes.

### Recommendations

We recommend that an updated test guideline for the mysid chronic toxicity study follow the format used in the other USEPA Series 850 Test Guidelines issued in 2016 (https://www.epa.gov/test-guidelines-pesticides-and-toxic-substances/series-850-ecological-effects-test-guidelines), including a clear list of the response variables to be calculated and evaluated and a listing of test validity elements (to include only those elements that would be expected to lead to study rejection). In addition, an updated test guideline should consider improvements that have been made over time in the culturing of mysids, feeding regimens, environmental conditions, and other test design factors.

To improve the draft OPPTS test guideline, we recommend the following criteria related to mysid survival and reproduction.

Based on control data collected from over 100 chronic mysid toxicity studies, the draft OPPTS test guideline acceptability criterion of three young produced per female per day is unreasonably restrictive and is unachievable. None of the control groups in the data set meet this criterion. The ASTM International test guideline acceptability criterion of an average of three young produced per female over the test period is more appropriate and is supported by the control data set, with all control groups associated with average numbers of young per surviving female greater than three.

The draft OPPTS test guideline does not currently include acceptability criteria for survival, while the ASTM International test guideline includes a criterion of 70% adult mysid survival. Based on the data set, an average of at least 70% adult survival was achieved by control groups in 93% of the studies. This additional criterion appears to provide a reasonable threshold for control performance and should be included in a test guideline update.

The draft OPPTS test guideline requests length be reported at sexual maturity and test termination. However, only a minority of studies report length at sexual maturity. The lack of reporting of length at sexual maturity may be due to the challenges of measuring mysid length prior to test termination without adversely impacting the test organism. We recommend removing this reporting requirement. Growth effects can continue to be captured by the measurement at test termination without the risk of impacting the test organism mid-exposure.

The draft OPPTS test guideline does not include acceptability criteria related to G2 measurement endpoints but does include reporting criteria. Survival of G2 organisms is consistently high, while other measurement endpoints, including the number of males and females and body length by sex, are rarely reported (<20% of studies). The review of the chronic mysid final study reports indicates that NOECs are so rarely based on G2 measurement endpoints that inclusion of G2 exposures is unnecessary. Omitting the G2 exposures would also make this guideline more similar to those for chronic toxicity tests with other invertebrates, such as *Daphnia*.

## Conclusion

Valid completion of the mysid chronic toxicity study can be challenging because the current (but 27-year-old) draft OPPTS test guideline provides limited technical information on study design and study conduct. The draft OPPTS test guideline states that the test should be rejected if either more than 25% of females fail to produce young or less than three offspring are produced per female per day. In addition to performance criteria for the control groups, the draft OPPTS test guideline includes reporting requirements for several measurement endpoints, including mysid body length by sex at sexual maturity, mysid body length by sex at test termination, and G2 mysid survival and body length by sex. Some of these reporting requirements are considered unlikely to have a significant effect on the test outcome based on the data analysis conducted herein.

Following review of the draft OPPTS test guideline and comparison with the ASTM International test guideline, and analysis of control data from over 100 studies, the recommendations of the CLA-CLE working group are: (1) Change the acceptability criterion for control reproduction from an average of three young per female per day to three young per surviving female. This is consistent with the ASTM International test guideline. (2) Add an acceptability criterion of 70% for control survival, consistent with the ASTM International test guideline. (3) Remove measurement of length at sexual maturity; length and weight can be measured at test termination. (4) Eliminate the requirement to conduct G2 exposures. The G2 exposures are optional under the ASTM International test guideline.

## Supplementary Material

vgaf036_Supplementary_Data

## Data Availability

The summary of the data used for the analysis is provided in the online supplementary material. The individual studies are coded with respect to identity of the pesticide, sponsor, performing laboratory, and other details. These details for the individual studies are considered Confidential Business Information and cannot be made publicly available.
